# Predictive imaging for tumor response to drug-eluting microsphere transarterial chemoembolization in patients with BCLC-C advanced hepatocellular carcinoma

**DOI:** 10.1038/s41598-019-56545-1

**Published:** 2019-12-27

**Authors:** Kai-Hsiang Chang, Zhen-An Hwang, Ping-Ying Chang, Hsuan-Hwai Lin, Yu-Lueng Shih, Wei-Chou Chang, Guo-Shu Huang, Hsian-He Hsu

**Affiliations:** 1 Department of Radiology, Tri-Service General Hospital, Taipei, Taiwan, Republic of China; 2 School of Medicine, National Defense Medical Center, Taipei, Taiwan, Republic of China; 3 Department of Radiology, Wan Fang Hospital, Taipei Medical University, Taipei, Taiwan, Republic of China; 4 Division of Gastroenterology, Department of Internal Medicine, Tri-Service General Hospital, Taipei, Taiwan, Republic of China; 5 Division of Hematology and Oncology, Department of Internal Medicine, Tri-Service General Hospital, Taipei, Taiwan, Republic of China

**Keywords:** Hepatocellular carcinoma, Cancer imaging, Chemotherapy

## Abstract

Drug-eluting microsphere transarterial chemoembolization (DEM-TACE) has been introduced to ensure more sustained and tumor-selective drug delivery for permanent embolization of HCC. The aim of this study was to determine the imaging characteristics that related to favourable treatment response in BCLC-C HCC patients treated with DEM-TACE. In total, 64 patients with BCLC-C HCC that treated with DEM-TACE using doxorubicin-eluted microspheres were retrospectively included. The images were assessed at baseline and at 4–12 weeks follow-up after receiving DEM-TACE. Pre- and post-procedural imaging characteristics were analysed by two independent radiologists and treatment response was evaluated using the modified Response Evaluation Criteria in Solid Tumors criteria. Multivariate analysis showed that vascular lake phenomenon (OR = 5.94, p = 0.03*), and homogeneous tumor enhancement (HTE) on cone-beam computed tomography (CBCT) during angiography (OR = 11.66, p < 0.001*) are associated with better radiological response. In contrast, residual tumor blush (OR = 0.11, p < 0.001*) is associated with worse radiological response. In conclusion, the initial tumor burden <50% (p = 0.012*) and HTE on CBCT (p = 0.040*) are good predictors for locoregional tumor control in patients with advanced HCCs, which can potentially improve patients’ outcome.

## Introduction

Hepatocellular carcinoma (HCC) is an aggressive malignancy and the fourth leading cause of cancer-related death each year^1^. Despite the recognition of cirrhosis as the major risk factor for HCC, more than 50% of patients with HCC present an advanced disease at diagnosis^2,3^. The concept of “advanced” disease varies considerably in several staging systems because the prognosis of an individual advanced HCC patient depends on not only tumor size, biologic behavior and spread of the tumor, but also on the degree of functional failure of the liver due to the presence of cirrhosis. By the Barcelona Clinic Liver Cancer (BCLC) definition^4–6^, advanced HCC is considered as an unresectable HCC with one of the following condition: extrahepatic spread (metastases or lymph nodes involvement), vascular invasion, or cancer-related symptoms (performance status 1–2). The prognosis in these patients are generally poor, with a reported median overall survival of 11 months.

Conventional lipiodol-based transarterial chemoembolization (cTACE) is generally accepted as a palliative treatment for unresectable HCC, but it is not officially included in the BCLC algorithm in advanced disease because of the possible adverse events, such as acute hepatic decompensation, especially in patients with portal vein tumor thrombus (PVTT). Not only systemic toxicity, but also the lack of standardization of the technique are the major limitations of cTACE. The emulsification of the chemotherapeutic drug and lipiodol is prepared extemporaneously and hence is operator dependent. Furthermore, the interpretation of post-treatment computed tomography (CT) of cTACE is sometimes difficult. The scattering and heterogeneity caused by intratumoral lipiodol deposition, especially in large HCCs, frequently obscure the viable tumor.

Non-resorbable embolic microspheres loaded with cytotoxic drugs has been developed to overcome these major drawbacks. Drug-eluting microsphere TACE (DEM-TACE) allows a better intratumoral repartition and improved pharmacokinetic profile, therefore achieving steady release of chemotherapeutic drug^7^. Several studies have addressed survival benefits in patients with advanced HCC, non-inferior treatment outcomes^8–11^ and safety profile versus sorafenib and/or cTACE^10,12–16^. However, to date, the predictive imaging for tumor response to DEM-TACE in patients with BCLC-C advanced HCC has never been discussed.

In order to understand imaging features in patients with advanced HCC who underwent DEM-TACE treatment, the purpose of this study is to evaluate the periprocedural imaging features during DEM-TACE and to find the imaging predictors for tumor response.

## Methods

### Ethics and study population

**Figure 1:**
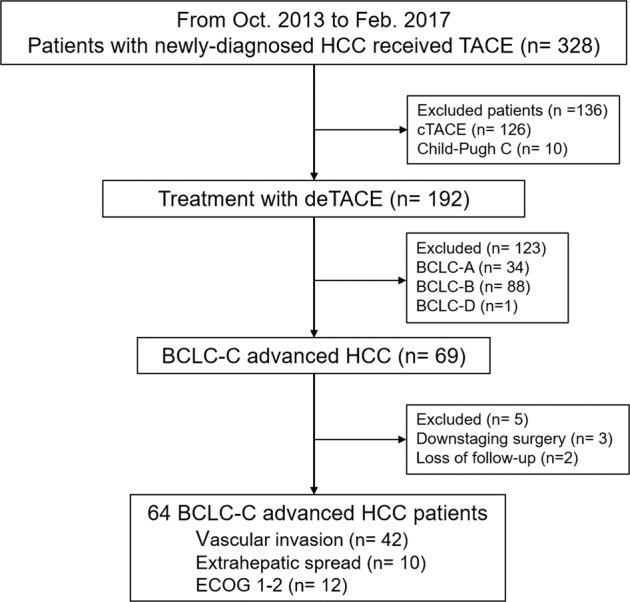
Patient selection criteria algorithm.

A computerized search of patients with newly diagnosed HCC treated by TACE was performed in Tri-Service General Hospital, Taipei, Taiwan from October 2013 to February 2017. The Institutional Review Board of Tri-Service General Hospital, National Defense Medical Center (TSGHIRB) approved the study and waived the informed consent requirement owing to the investigation’s retrospective nature. Patients who met the following criteria were included: (1) unresectable HCC confirmed by surgeons including bilobular involvement of the liver and invasion of major blood vessels such as main portal vein, hepatic veins, inferior vena cava, and main hepatic artery etc.; (2) BCLC-C HCC was confirmed either based on dynamic CT/MRI imaging or Eastern Cooperative Oncology Group (ECOG) status; and (3) patients had at least one follow-up CT/MRI image. Patients with Child-Pugh C cirrhosis, poor data integrity, obstructive jaundice, or uncorrectable hepatic encephalopathy were excluded. A total of 328 consecutive patients with newly diagnosed HCC were reviewed. Among all, 126 patients receiving cTACE and 10 patients with Child-Pugh C disease were excluded. Eventually, 192 patients who received DEM-TACE as initial treatment for unresectable HCC were included. The patient selection process is shown in Fig. 1.

### Chemoembolization procedure and regimen

Eighteen (28.1%) patients received sequential or combination therapy with oral Sorafenib (Nexavar®, Bayer AG, Leverkusen, Germany), and most of these patients (n = 14, 77.8%) began the treatment course within 1 month prior to DEM-TACE treatment. The starting dose of oral Sorafenib was 400 mg twice daily until disease progression or patient refusal.

Patients were admitted one day before the procedure for preprocedural evaluations and under nil per oral status for at least 8 hours. Each vial of HepaSpheres^TM^ (Merit Medical Systems, Inc) were loaded with 50 mg powder-form of Doxorubicin hydrochloride following the manufacturer’s instructions and yielded 30 ml of mixed chemo-embolic emulsion. The maximal dosage of embolic agents is one vial for small HCCs (size < 5 cm) and two vials for large HCCs (size > 5 cm). When two vials of Hepaspheres were not sufficient to complete embolization for large HCC with plenty of tumor vascularity, further embolization with Gelfoam® sponge would be performed until reaching the sub-stasis angiographic endpoint. TACE were performed using a 5.0 Fr. catheter via right femoral artery. Routine celiac and superior mesenteric arteriography were performed for assessing the arterial anatomy, tumor supply, anatomical variants, and patency of the portal vein. Tumor-feeding arteries were cannulated superselectively using microcatheters and embolized with doxorubicin-eluting microspheres. Additional angiography was performed to detect the extrahepatic blood supply. If present, extrahepatic arterial supplies were also superselectively cannulated and embolized with drug-eluting microspheres until the embolization endpoint.

The embolization endpoint was sub-stasis of antegrade arterial flow and complete devascularization of the target lesions. The completion of chemoembolization depends upon the angiographic endpoint decided by the interventional radiologist in charge of the procedure. In addition, if vascular lake phenomenon (VLP) was observed during the chemoembolization, the procedure continued unless the patient experienced severe abdominal discomfort or the VLP showed extra-capsular leakage. In such conditions, further embolization with Gelfoam^®^ sponge to occlude the major tumor supplying artery would be performed.

### Imaging parameters evaluation

Images were assessed at baseline and at 12 weeks follow-up after DEM-TACE. Among 64 patients, 42 patients underwent CT (65.6%) and 22 patients underwent MRI (34.3%). The pre-procedural and post-procedural images were done using either four-phase dynamic CT or MR and the same imaging modalities were required for post-procedural follow-up imaging, to avoid the inconsistency in mRECIST tumor response evaluation. The timing for performing pre-procedural baseline imaging was within 4 weeks prior to DEM-TACE treatment; and post-procedural follow-up imaging was performed between 4 to 12 weeks after DEM-TACE treatment.

**Figure 2:**
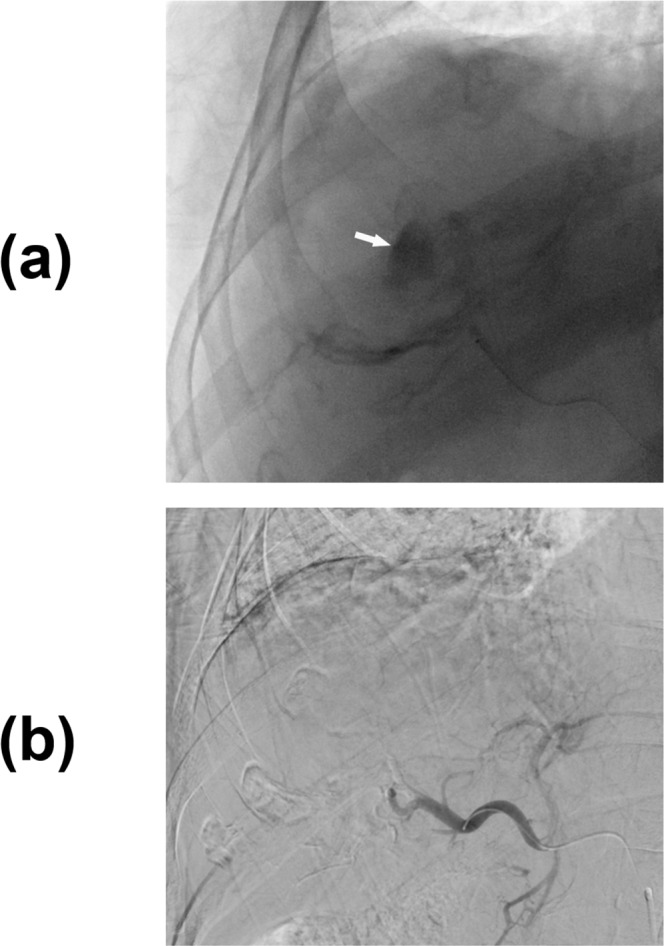
Right hepatic angiogram shows a (**a**) vascular lake that presents as localized contrast pooling (white arrow) during angiography. (**b**) Superselective angiogram shows disappearance of the vascular lakes, and no contrast extravasation was observed.

**Figure 3:**
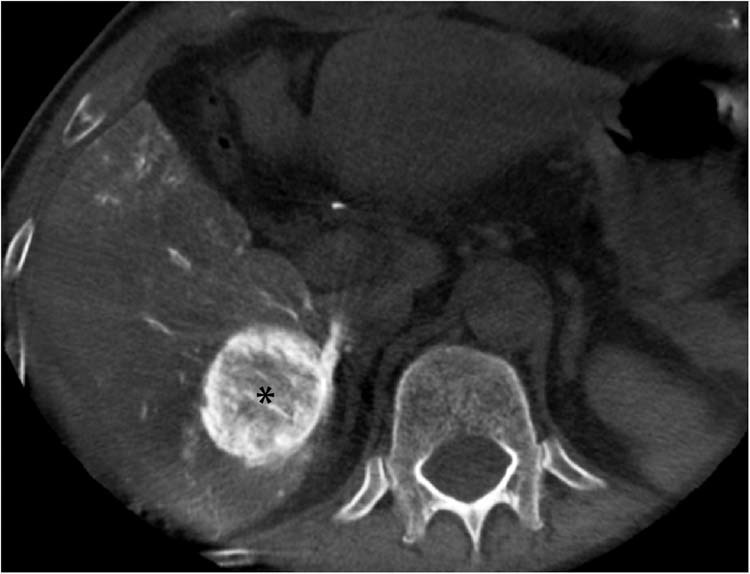
Contrast-enhanced cone-beam CT in the axial plane demonstrates homogeneous tumor enhancement and evenly distributed contrast medium within the tumor (asterisk).

**Figure 4:**
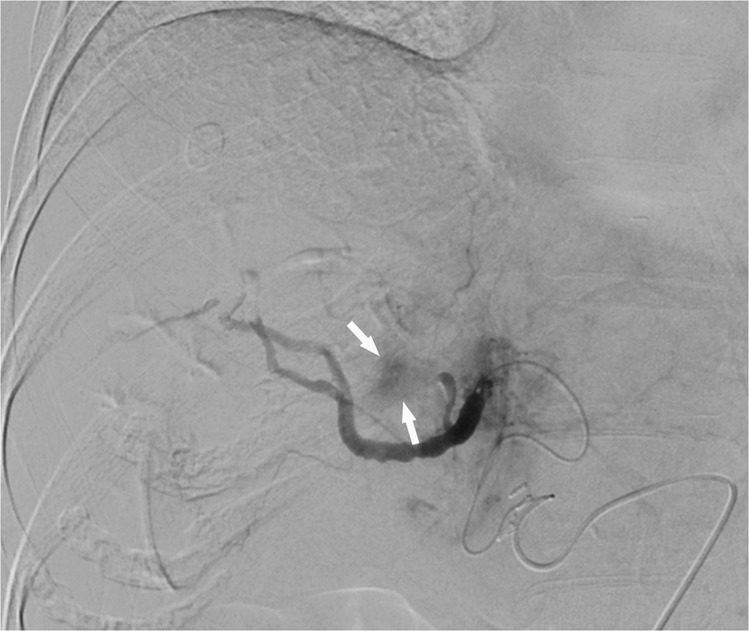
Post-TACE right hepatic angiogram showing residual tumor blush (white arrows) during arterial-phase imaging. It is a residual intratumoral enhancement on completion arteriography during TACE.

The periprocedural angiographic images were interpreted by two independent diagnostic radiologists using the same imaging protocol as the baseline. To standardize the measurements, definition and quantitative measurement for each parameter were discussed before image interpretation to reach consensus. The individual image reviews by both readers were used to calculate interobserver variability. The definitions of baseline CT or MR images were reviewed as follows: (1) Arterial-phase hyperenhancement. (2) Washout in portal-venous phase or delayed phase. (3) Enhancing capsule, defined as an enhanced, sharp border surrounding the mass that persisted in the portal-venous phase or delayed phase. (4) Tumor morphology: classified as ill-defined or well-defined margins. (5) Cystic necrosis or degeneration, defined as present if larger than 50% of the tumor volume. (6) Macroscopic vessel involvement, defined as direct invasion or thrombus of the portal vein, hepatic vein, or inferior vena cava detected macroscopically. (7) Exophytic growth, defined as outward growth beyond the capsule. (8) Intrahepatic vascular shunt: showing peri-tumoral transient hepatic attenuation differences or early portal vein enhancement in the arterial phase before enhancement of the main portal branches. Perioperative angiographic findings were evaluated with the following definitions: (1) Largest feeding artery diameter was measured at the entry site of each vessel into each target lesion and compared to the inner diameter of the microcatheter on a case-by-case basis. (2) Number of tumor-supplying first-order branches of the celiac trunk, superior mesenteric artery and in some cases, directly from abdominal aorta. (3) Vascular lake phenomenon (VLP), defined as localized intra-tumoral contrast accumulation during angiography when injecting microspheres into the tumor. (Fig. 2) (4) Homogeneous tumor enhancement (HTE), defined as uniform contrast distribution without missing any corner of the target lesion during the arterial-phase scan of superselective, preprocedural cone-beam CT (CBCT) (Fig. 3). (5) Residual tumor blush, which presents as residual intra-tumoral enhancement on completion arteriography during TACE (Fig. 4).

### Evaluation of treatment response after DEM-TACE

Patients were followed up at an interval of 12 weeks if there was no evidence of viable tumor or newly developed lesions. Treatment responses were evaluated by another abdominal diagnostic imaging specialist with 12-year experience, using the modified Response Evaluation Criteria in Solid Tumors (mRECIST) criteria and further categorized target lesions as complete response (CR), partial response (PR), stable disease (SD) and progressive disease (PD). CR or PR lesions were considered responders, whereas nonresponders were defined as SD or PD lesions.

### Endpoint and statistical analysis

Overall survival (OS) was measured from DEM-TACE initiation until the end of the study or death. Survival endpoints were estimated using the Kaplan-Meier method. The response for each treated tumor was evaluated using mRECIST criteria. The objective response rate (ORR) and disease control rate (DCR) after each DEM-TACE procedure were calculated according to mRECIST criteria. If the patient had multinodular disease, the classification as a responder or nonresponder was determined using the largest target lesion.

Statistical analysis was performed using SPSS software (SPSS for Windows, version 20.0). To determine the significance of differences between the responder and nonresponder groups, chi-square test was used, and differences were considered significant when p < 0.05. The results are expressed as the mean value ± standard deviation for continuous variables and as the absolute frequency and percentage for categorical variables. OS was analysed using the Kaplan-Meier method. The log-rank test was used to analyse prognostic factors. The imaging variables were first evaluated by univariate analysis and further evaluated by multivariate analysis if they were statistically significantt. Intra-observer variability was not estimated, as each radiologist assessed the CT or MRI images once only. Interobserver variability of the imaging parameters was estimated using Cohen’s *kappa* coefficient (κ) and was classified as follows: slight = 0–0.20; fair = 0.21–0.40; moderate = 0.41–0.60; substantial = 0.61–0.80; and almost perfect = 0.81–1.00.

## Results

### Baseline demographics and characteristics

In total, 64 patients with 86 HCC target lesions were included, consisting of 42 (65.6%) men and 22 (34.3%) women with a mean age of 61.3 ± 12.4 (mean ± SD) years. The demographics, underlying diseases and tumor characteristics are listed in Table 1. The most predominant etiology was hepatitis B virus infection (57.8%), followed by multifactorial or indistinguishable (28.1%) and hepatitis C virus infection (14.1%). Forty-four patients were classified as Child-Pugh class A (68.7%), and the remaining 20 patients were classified as Child-Pugh class B (31.3%). 78.1% of the patients (50 of 64) had a tumor burden <50%, and 21.9% (14 of 64) had a tumor burden >50%. The mean size of the target lesions (cm) was 6.7 ± 4.0 (range, 1.2–20.3). Forty-two patients (65.6%) presented with intrahepatic vein invasion, in which portal vein invasion accounted for 76.2% (32 of 42), systemic venous invasion was observed in 7.1% (3 of 42), and 16.7% (7 of 42) had both. 10 of 64 patients (15.6%) exhibited extrahepatic spread, including nodal metastasis and distant metastasis. Among all the patients, 12 of 64 (18.7%) had ECOG 1–2, and 28.1% of (18 of 64) received combined therapy with oral Sorafenib. α-fetoprotein (AFP) elevation was seen in 39 patients (60.9%). The mean hospital stay was 9.6 ± 6.5 days. The average infused volume of mixed chemo-embolic agent was 40.78 ± 15.46 (mean ± SD) ml with a mean dosage of 67.96 ± 25.77 (mean ± SD) mg of doxorubicin.

**Table 1:** Patients Demographics.

Categories	Incidence/Value
Age (years)	61.3 ± 12.4 (27–85)
Gender	
Female	22 (34.3%)
Male	42 (65.6%)
Etiology of liver disease	
HBV	37 (57.8%)
HCV	9 (14.1%)
Multifactorial/ others	18 (28.1%)
Child-Pugh class	
A	44 (68.7%)
B	20 (31.3%)
Underlying disease	
Diabetes	25 (39.1%)
Hypertension	15 (23.4%)
Renal function impairment	6 (9.3%)
Tumor burden	
>50%	14 (21.9%)
<50%	50 (78.1%)
Frequencies of DEM-TACE treatments	2.17 (1–5)
Pre-DEM-TACE AFP level	
<20	25 (39.1%)
>20	39 (60.9%)
Size of target lesion (cm) before DEM-TACE	6.7 ± 4.0 (1.2–20.3)
Sorafenib usage	18 (28.1%)
Intrahepatic vein invasion (n = 42)	42 (65.6%)
Portal vein	32/42 (76.2%)
Systemic vein	3/42 (7.1%)
Both	7/42 (16.7%)
Extrahepatic Spread	10 (15.6%)
ECOG	
0	52 (81.3%)
1–2	12 (18.7%)

Data are expressed as mean standard deviation for continuous variables. Other data are expressed as number of patients(percentage) for categorical variables. HBV: Hepatitis B virus; HCV: Hepatitis C virus; DEM-TACE: Drug-eluting transarterial chemoembolization; BCLC: Barcelona Clinic Liver Cancer; ECOG: Eastern Cooperative Oncology Group.

### Treatment response and subgroup analysis between responders and nonresponders

31.4% (n = 27) of patients achieved CR, 26.7% (n = 23) had PR, 30.2% (n = 26) had SD, and 11.6% (n = 10) had PD. The 3-month overall objective response was 58.1% (50 of 86). Responders accounted for 58.1% (n = 50) of all target lesions, and the remaining 41.9% (n = 36) were nonresponders. Table 2 details the treatment responses of the target lesions.

**Table 2:** Tumor response and overall survival of BCLC-C advanced HCC patients.

	Tumor response rate (per target lesion) at 1^st^ follow-up within 3 months
Complete Response	27/86 (31.4%)
Partial Response	23/86 (26.7%)
Stable Disease	26/86 (30.2%)
Progressivea Disease	10/86 (11.6%)
Objective Response	58.1%
Disease Control Rate	88.4%
**OS/person (per patient)**	
Median OS (months)	15.9 ± 8.7
1-year OS rate	38/64 (59.3%)
2-year OS rate	14/64 (21.8%)

OS: Overall Survival, BCLC: Barcelona Clinic Liver Cancer.

Table 3 summarizes the imaging characteristics and analysis comparing responders and nonresponders. In the preprocedural imaging analysis, the presence of intrahepatic vascular shunts is the only factor that contributed to a more intractable tumor response (responders: 15 of 50 [30.0%]; nonresponders: 22 of 36 [61.1%], *p* = 0.004*). Arterial-phase hyperenhancement (*p* = 0.982), washout on portal-venous or delayed phase (*p* = 0.400), tumor capsule (*p* = 0.421), tumor morphology (*p* = 0.063), cystic degeneration (*p* = 0.657), macroscopic vessel involvement (*p* = 0.595) and exophytic tumor growth (*p* = 0.803) were not statistically significant. The presence of VLP (*p* = 0.037*), the absence of residual tumor blush (*p* < 0.001*) and HTE on CBCT before the DEM-TACE procedure (*p* < 0.001*) were significantly associated with a better radiological response in the univariate analysis (Table 4). In the multivariate analysis, only the absence of residual tumor blush (*p* < 0.001*), the presence of VLP (*p* = 0.030*) and HTE (*p* < 0.001*) were found to be independent predictors of a better radiological response. Table 4 summarizes the univariate and multivariate logistic regressions between responders and nonresponders and the variables associated with a more promising radiological tumor response.

**Table 3:** Pre-procedural Imaging Characteristics between Responders and Non-responders.

	Responder (n = 50)	Non-responder (n = 36)	*P* value
Sorafenib usage	13 (32.5%)	5 (20.8%)	0.312
Pre-procedural Imaging Characteristics			
APHE			0.982
Absent	14 (28.0%)	10 (27.8%)	
Present	36 (72.0%)	26 (72.2%)	
Washout on portal venous phase			0.400
Absent	10 (20.0%)	10 (27.8%)	
Present	40 (80.0%)	26 (72.2%)	
Capsule			0.421
Absent	35 (70.0%)	28 (77.8%)	
Present	15 (30.0%)	8 (22.2%)	
Tumor morphology			0.063
Well-defined	41 (82.0%)	23 (63.9%)	
Infiltrative	9 (18.0%)	13 (36.1%)	
Cystic degeneration			0.657
Absent	31 (62.0%)	24 (66.7%)	
Present	19 (38.0%)	12 (33.3%)	
Macroscopic vessel involvement			0.595
Absent	14 (28.0%)	12 (33.3%)	
Present	36 (72.0%)	24 (66.7%)	
Exophytic growth			0.803
Absent	40 (80.0%)	28 (77.8%)	
Present	10 (20.0%)	8 (22.2%)	
Intra-hepatic vascular shunt			0.004*
Absent	35 (70.0%)	14 (38.9%)	
Present	15 (30.0%)	22 (61.1%)	
Angiographic Characteristics			
Feeding artery diameter			0.865
≤microcatheter	9 (18.0%)	7 (19.4%)	
>microcatheter	41 (82.0%)	29 (80.6%)	
Number of supplying artery*			0.934
Single	42 (84.0%)	30 (83.3%)	
Multiple	8 (16.0%)	6 (16.7%)	
Vascular lake phenomenon (VLP)			0.037*
Absent	35 (70.0%)	32 (88.9%)	
Present	15 (30.0%)	4 (11.1%)	
HTE on CBCT			<0.001*
Yes	40 (80.0%)	6 (16.7%)	
No	10 (20.0%)	30 (83.3%)	
Residual tumor blush			<0.001*
No	28 (56.0%)	3 (8.3%)	
Yes	22 (44.0%)	33 (91.7%)	
Sub-stasis endpoint			0.720
No	4 (8.0%)	5 (13.9%)	
Yes	46 (92.0%)	31 (86.1%)	

Data are expressed as number of target lesions(percentage). P values derived from the chi-square test are significant (P < 0.05*) HTE: homogeneous tumor enhancement; CBCT: cone-beam CT; DEM-TACE: Drug-eluting transarterial chemoembolization.

**Table 4:** Univariate and multivariate logistic regression associating with tumor response.

Variables	Tumor responders/target lesions (%)	Univariate		Multivariate	
		Odds ratio	*P* value*	Odds ratio	*P* value*
HTE on CBCT	40/46 (86.9%)	20.0	<0.001*	11.66	<0.001*
Residual tumor blush	22/55 (40.0%)	0.07	<0.001*	0.11	<0.001*
Vascular lake phenomenon	15/19 (78.9%)	3.43	0.037*	5.94	0.030*
Intrahepatic vascular shunts	15/37 (40.5%)	0.27	0.004*	0.31	0.074

HTE: Homogeneous tumor enhancement; CBCT: cone-beam CT.

### Survival outcome analysis

**Figure 5:**
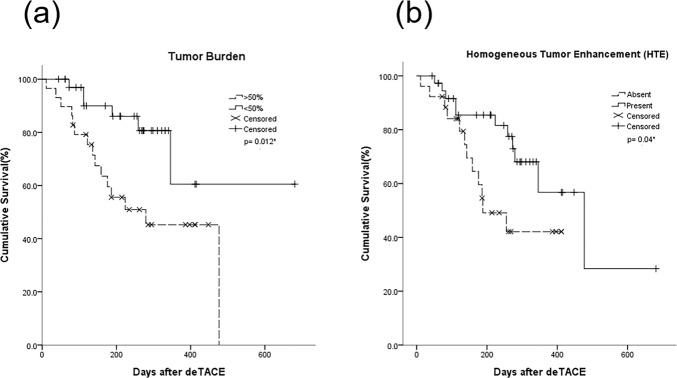
Kaplan–Meier survival curves assessing the median OS by comparing (**a**) Tumor burden, (**b**) Homogeneous tumor enhancement (HTE).

**Figure 6:**
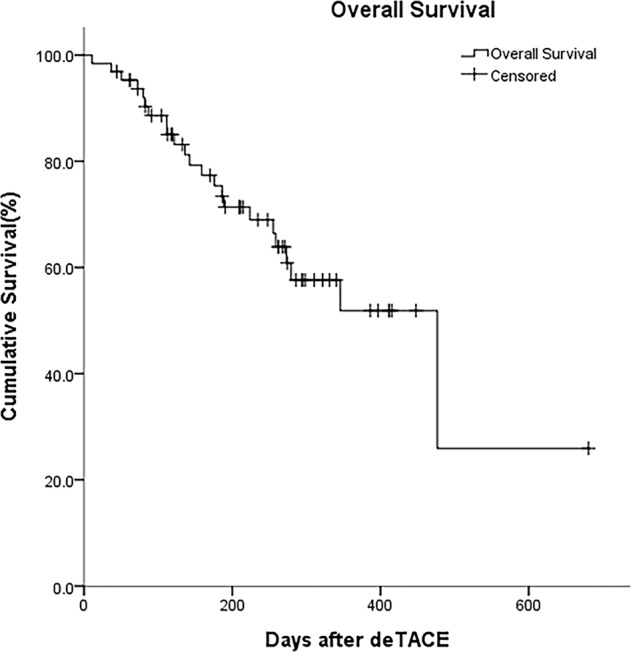
Kaplan–Meier estimates of overall survival.

The median OS was 15.9 ± 8.7 months, and the 1-year OS was 59.3%. In the subgroup analysis, patients with a tumor burden > 50% had a shorter median OS (279.5 days) compared to those with a tumor burden <50% (512.8 days) (*p* = 0.012*) (Fig. 5a). For patients with HTE on CBCT, median OS was 427.5 days, and those without HTE enhancement on CBCT had a median OS of 249.4 days (*p* = 0.040*) (Fig. 5b). Likewise, the median OS of responders and nonresponders was 512.8 days and 290.4 days, respectively (*p* = 0.015*). Patients with VLP had a median OS of 403.1 days, which was slightly but not significantly longer than those without VLP (293.6 days) (*p* = 0.618). The median OS of patients with intrahepatic vascular shunts and those without was 355.6 days and 410.8 days, respectively (*p* = 0.594). Other findings including macroscopic vascular invasion and residual tumor blush after DEM-TACE were not statistically significant. The Kaplan-Meier estimated survival curves are shown in Figs. 5 and 6.

### Interobserver agreement

The interobserver analysis was conducted using κ. Arterial-phase hyperenhancement (k = 0.77), enhancing capsule (k = 0.62), tumor morphology (k = 0.78), cystic necrosis/degeneration (k = 0.74), macroscopic vascular invasion (k = 0.61), feeding artery diameter greater than microcatheter inner diameter (k = 0.61) and HTE (k = 0.71) reached substantial agreement. The other features, including washout on portal-venous phase or delayed phase (k = 0.52), exophytic growth (k = 0.57), intrahepatic vascular shunts (k = 0.49), number of feeding artery(s) (k = 0.41), and the VLP (k = 0.48), showed moderate inter-rater agreement.

## Discussion

With the advancement of intravascular devices and microspheres, the delivery of chemotherapeutic agents can be accomplished more efficiently nowadays. In this retrospective study, DEM-TACE had shown an encouraging efficacy in treating BCLC-C patients. The response rate, including 3-month ORR of 58.1%, and CR rate of 54% were comparable to a previous report that mainly included BCLC-B, less advanced patients^14^. The survival outcomes, including median OS of 15.9 months, and 1-year OS of 59.3%, are also comparable to previous reports.

Post-DEM-TACE imaging characteristics exhibit some intriguing features providing real-time intraoperative response prediction and perspectives on future treatment planning. In this study, the multivariate analysis demonstrated that VLP, lack of residual tumor blush and HTE on CBCT were independent factors for better locoregional response; and tumor burden <50% and HTE on CBCT were factors associated with longer survival.

VLP is a common angiographic finding that possibly triggered by rupture of the tumor microvasculature, hence causing partial disruption of the tumor architecture and forming new blood spaces. Consistent with recent findings, this study demonstrated that VLP was an independent prognostic factor of a better tumor response^17,18^ but failed to demonstrate survival benefits in this study. HTE on CBCT is associated with better radiological response and higher OS rate. One possible explanation is that chemotherapeutic agents are more evenly distributed inside the tumors thereby ensuring more stable intratumoral drug concentration. A similar trend was observed by Kawamura *et al*. and Shimizu *et al*.^19,20^, who stated that HCC with HTE after RFA treatment was associated with a lower recurrence rate and mortality^19^. They concluded that a heterogeneous enhancement pattern is linked to poor histopathological differentiation, which has 28-times-higher likelihood of recurrence^19,21^. The association between enhancement pattern and histopathological differentiation stands another possible hypothesis. El-Assal *et al*. and Asyama *et al*. described diminished arterial blood flow in poorly differentiated and larger HCCs (>5 cm)^22,23^. They conjectured that rapid cell proliferation in the tumor centre increases the interstitial pressure, leading to compressive closure of tumor capillaries and regression of neovascularization, hence becoming hypovascular on imaging study. Consequently, a homogeneous enhancement pattern is more likely representing less aggressiveness, resulting in a better tumor response and longer survival. Interestingly, another 3D CT texture analysis using C + + language-based software by Park *et al*. stated that lower homogeneity was a significant predictors for CR after cTACE^24^, which seems to conflict with the aforementioned results. We believed this discordance maybe due to different definition of homogeneity or way of evaluation eg simple visual estimation or computerized CT attenuation measurement. Further investigation on HTE and clinical outcome will bring more insight on tumor prognosis.

Several limitations still exist in this study. First, it was a retrospective analysis conducted in a single institution and consisting of small patient number (n = 64). And, the follow-up interval ranged from 4 to 12 weeks, which might cause potential discrepancy in response evaluation. Future prospective studies are warranted for validation of our results and reduce probable bias. Second, we did not perform dose-tumor response relationship since there are many confounding factors leading to significant variability in how the procedure is performed, patient tolerability and the operator-dependent endpoints. As a loco-regional therapy, Jin *et al*.^25^ has found the intermediate, sub-stasis angiographic endpoint improved patient’s survival compared to embolization with a higher, stasis endpoint. The proper dose selection should follow a multifactorial decision process. Future randomized, dose-ranging studies are warranted. Third, the use of Sorafenib may obscure the DEM-TACE response. A total of 18 patients received sequential or combination therapy with oral Sorafenib in our study, and most of these patients (n = 14) began the treatment course within 1 month prior to DEM-TACE. It was believed that sorafenib may have influence on our results. Fourth, classifying angiographic findings based on DSA is intrinsically subjective and observer dependent. To overcome this limitation requires objective approaches to quantifying 3D computed analysis of angiographic characteristics. Lastly, the outcome of advanced HCC is determined by multiple factors and good radiological response along does not necessarily prolong patients’ survival. It is essential not to overemphasize imaging clues but view DEM-TACE as a potential combining treatment option for advanced HCC.

In conclusion, this study demonstrated that DEM-TACE is an effective treatment for BCLC-C advanced HCC. Furthermore, VLP, HTE on CBCT during angiography and tumor burden <50% are predictors for promising radiological tumor response and locoregional tumor control, which can potentially improve patients’ outcome.

## Data Availability

All data generated or analysed during this study, that are not included in this published article (and its Supplementary Information Files), are available from the corresponding author on reasonable request.
